# Spatial Localisation of Actin Filaments across Developmental Stages of the Malaria Parasite

**DOI:** 10.1371/journal.pone.0032188

**Published:** 2012-02-28

**Authors:** Fiona Angrisano, David T. Riglar, Angelika Sturm, Jennifer C. Volz, Michael J. Delves, Elizabeth S. Zuccala, Lynne Turnbull, Chaitali Dekiwadia, Maya A. Olshina, Danushka S. Marapana, Wilson Wong, Vanessa Mollard, Clare H. Bradin, Christopher J. Tonkin, Peter W. Gunning, Stuart A. Ralph, Cynthia B. Whitchurch, Robert E. Sinden, Alan F. Cowman, Geoffrey I. McFadden, Jake Baum

**Affiliations:** 1 The Walter and Eliza Hall Institute of Medical Research, Parkville, Victoria, Australia; 2 Department of Medical Biology, University of Melbourne, Parkville, Victoria, Australia; 3 School of Botany University of Melbourne, Parkville, Victoria, Australia; 4 Department of Biological Sciences, Imperial College of Science, Technology and Medicine, London, United Kingdom; 5 The ithree Institute, University of Technology Sydney, Sydney, New South Wales, Australia; 6 Department of Biochemistry and Molecular Biology, Bio21 Molecular Science and Biotechnology Institute, University of Melbourne, Parkville, Victoria, Australia; 7 Department of Pharmacology, School of Medical Sciences, University of New South Wales, Sydney, New South Wales, Australia; Weill Cornell Medical College, United States of America

## Abstract

Actin dynamics have been implicated in a variety of developmental processes during the malaria parasite lifecycle. Parasite motility, in particular, is thought to critically depend on an actomyosin motor located in the outer pellicle of the parasite cell. Efforts to understand the diverse roles actin plays have, however, been hampered by an inability to detect microfilaments under native conditions. To visualise the spatial dynamics of actin we generated a parasite-specific actin antibody that shows preferential recognition of filamentous actin and applied this tool to different lifecycle stages (merozoites, sporozoites and ookinetes) of the human and mouse malaria parasite species *Plasmodium falciparum* and *P. berghei* along with tachyzoites from the related apicomplexan parasite *Toxoplasma gondii*. Actin filament distribution was found associated with three core compartments: the nuclear periphery, pellicular membranes of motile or invasive parasite forms and in a ring-like distribution at the tight junction during merozoite invasion of erythrocytes in both human and mouse malaria parasites. Localisation at the nuclear periphery is consistent with an emerging role of actin in facilitating parasite gene regulation. During invasion, we show that the actin ring at the parasite-host cell tight junction is dependent on dynamic filament turnover. Super-resolution imaging places this ring posterior to, and not concentric with, the junction marker rhoptry neck protein 4. This implies motor force relies on the engagement of dynamic microfilaments at zones of traction, though not necessarily directly through receptor-ligand interactions at sites of adhesion during invasion. Combined, these observations extend current understanding of the diverse roles actin plays in malaria parasite development and apicomplexan cell motility, in particular refining understanding on the linkage of the internal parasite gliding motor with the extra-cellular milieu.

## Introduction

Malaria constitutes a huge health and economic burden on humanity [1]. The disease is caused by obligate intracellular parasites from the genus *Plasmodium*, a group of protozoa whose developmental lifecycle is completed between mosquito and human hosts. During this complex journey the parasites navigate a variety of tissues and infects several distinct cell types [2], [3], [4]. Three motile and/or invasive forms define this journey: ookinete, sporozoite and merozoite. The ookinete traverses the mosquito midgut [4]. The sporozoite establishes salivary gland infection in the mosquito along with subsequent transmission to the human host and infection of the liver [3]. Finally, the merozoite, the smallest form, infects circulating erythrocytes in the bloodstream and is responsible for initiating all pathology associated with malaria disease [2]. Despite gross morphological differences and disparate environmental niches, each of these developmental forms retain the classical cytoskeletal architecture and organelle repertoire (with the exception of the ookinete) of apicomplexan parasites [5], [6], the phylum to which malaria parasites belong. Furthermore, each retains a conserved way of moving and invading cells based on actin and myosin, termed gliding motility [7].

The current model for the gliding motor [8] is centred on a short single-headed myosin that is attached to a double membrane bound complex of organelles called the inner membrane complex (IMC), which lies directly under the plasma membrane [9]. Microfilaments of actin are then thought to form in the intervening space, called the supra-alveolar space, between IMC and plasma membrane [10]. On polymerisation these actin filaments are thought to provide the key rigid element upon which myosin bears to create the required rearward traction force for movement. The myosin stroke is then conveyed, via the actin filament, to surface bound adhesins from the thrombospondin-related anonymous protein (TRAP) family [11]. Coupling of TRAP to surface receptors in the extra-cellular milieu, transmits the internal rearward force, driving the parasite forwards [12]. The topology of this motor model is largely based on immunoprecipitation data along with immunofluorescence imaging of several core components in malaria parasites and the related apicomplexan parasite *Toxoplasma gondii* [8], [11], [13], [14].

According to the current model for gliding motility, actin filaments only form transiently at sites where the gliding motor is engaged – in other words, sites where traction is being applied between the parasite motor and the substrate surface or host cell [15]. In parasites that are focussed primarily on movement in the absence of true invasion, for example the ookinete [4], this could be anywhere along the length of the cell where the stage-specific TRAP-like adhesin is linked to the extra-cellular environment. For parasites that actively invade host cells, such as the sporozoite and merozoite, this would presumably be restricted to the tight junction – an electron dense interface formed between the invading parasite and its host cell [16]. Whilst the molecular architecture of the junction has been elucidated in great detail recently [17], its linkage to the actomyosin motor is currently unknown. Indeed, to date, and despite its essential role in motility, no study has provided direct visual evidence for the placement of actin filaments in the parasite pellicle of any moving apicomplexan cell [18], [19], [20], [21].

The evidence in support of actin's role in motility comes largely from actin inhibitors that disrupt parasite gliding and host cell invasion [15], [22], [23], [24]. Of these, the marine sponge cyclodepsipeptide Jasplakinolide (JAS) has proven particularly useful, binding to and stabilising formed filaments preventing disassembly [25]. Use of JAS has facilitated the demonstration of high concentrations of dynamic actin at the apex of free *Plasmodium* merozoites, ookinetes and *T. gondii* tachyzoites [21], [24], [26], [27]. Microfilament structures, presumed to be actin, have also been seen lying under the plasma membrane of motile tachyzoites following JAS treatment [28]. Complementing these studies, electron and cryo-electron microscopy studies have observed structures with dimensions consistent with filamentous actin in this pellicular compartment under native conditions [19], [20]. Beyond these encouraging observations, however, no study has unambiguously demonstrated microfilament spatial organisation during zoite movement under native conditions. This likely derives from the intrinsic short length of apicomplexan actin filaments (∼100 nm), their instability, dynamic and transient nature and the poor utility of conventional filament markers such as phalloidin with apicomplexan cells [29], [30], [31], [32].

Aside from motility actin likely plays several additional roles in parasite development, including roles in haemoglobin uptake [33] and general vesicular trafficking [34] along with several possible functions in the nucleus [35]. However, like motility, these roles have remained incompletely explored because of difficulties in decisively localising actin and its microfilaments within parasite cells. To visualize the spatial dynamics of malaria parasite actin we generated mouse and rabbit parasite-specific antibodies towards actin I (the conserved isoform implicated in most actin-dependent processes across Apicomplexa [36]) that recognises filamentous actin in preference to monomeric actin. We employed these tools on mouse and human malaria parasites to gain access to the three major motile or invasive lifecycle forms (ookinete, sporozoite and merozoite) along with asexual blood stages and tachyzoites from *T. gondii* to provide a map for dynamic actin filament formation. We demonstrate actin concentrates in discrete zones in the nuclear compartment during development, within the supra-alveolar space during motility, and at sites predicted to be core regions of traction during host cell invasion. These results point to new functions for actin in parasite development and refine current understanding of the role of microfilaments during key stages of parasite infection.

## Results

### Generation of a malaria parasite actin-specific antibody

**Figure 1:**
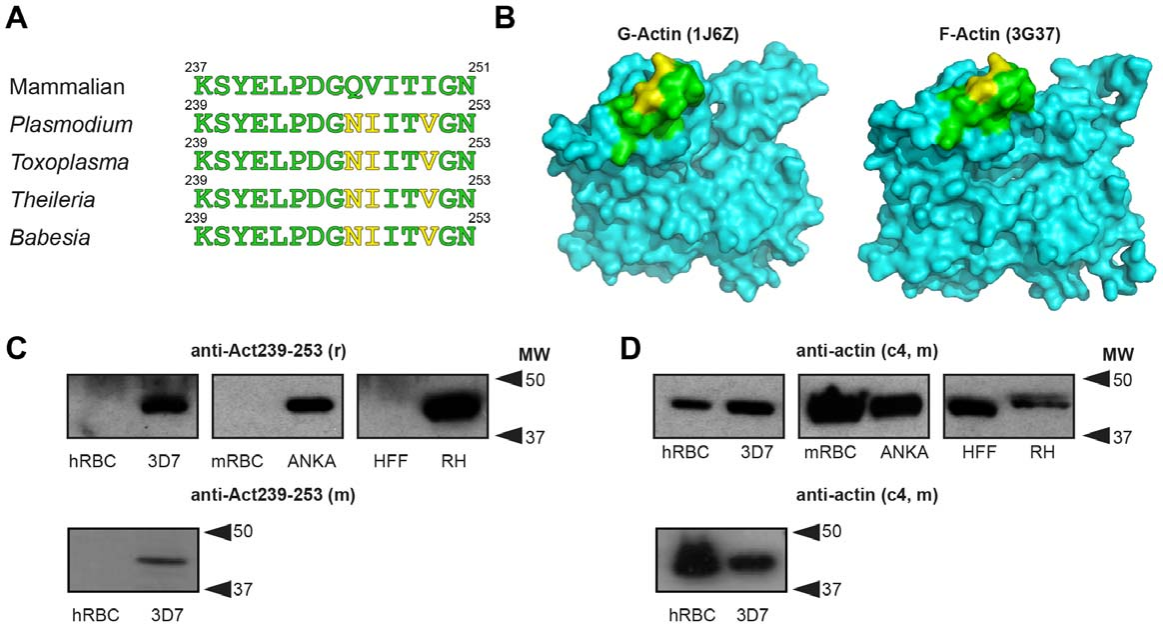
An apicomplexan parasite-specific anti-actin antibody. **A**) Sequence comparison between human non-muscle actin amino acids 237–251 (the basis of anti-Gly_245_ [39]) and apicomplexan actin I orthologues over the amino acids 239–253 (the basis for anti-Act_239–253_). **B**) Surface representation of the structures of rabbit G-actin (PDB:1J6Z; A) and a protomer in rabbit F-actin (PDB:3G37; B) showing anti-Gly_245_ epitope. Residues in yellow indicate polymorphisms between mammalian and *P. falciparum* actin. **C**) Representative immunoblot showing reactivity of rabbit® anti-Act_239–253_ serum with human erythrocytes (hRBC), asexual *P. falciparum* (3D7), mouse erythrocytes (mRBC), asexual *P. berghei* (ANKA), human foreskin fibroblasts (HFF) and *T. gondii* tachyzoites (RH). Lower panel shows same hRBC and 3D7 sample probed with mouse (m) anti-Act_239–253_ serum. **D**) As C but using generic anti-actin monoclonal C4.

Conventional antibodies against mammalian actin have been used successfully to label the entire actin pool in *Toxoplasma gondii* tachyzoites [37] and *Plasmodium* merozoites and ookinetes [22], [38]. However, these antibodies cannot differentiate monomeric (G)- from filamentous (F)- actin and have the added drawback of also recognising host cell actin with equal or greater affinity. Serum generated against a short peptide corresponding to amino acids 237–251 of non-muscle mammalian actin, anti-Gly_245_ [39] (Fig. 1A), has been reported to preferentially recognise short actin filament ends associated with vesicle transport in human fibroblasts [40]. This epitope, on sub-domain 4 of the actin monomer, is exposed in free actin monomers and at the end of the filamentous form (Fig. 1B). The specificity for short filament ends is thought to result from the epitope being hidden in the body of filaments (from subunit contact), long filament ends (as a result of capping) and in free monomers either by virtue of the topology of the epitope in monomers versus filaments (Fig. 1B) or because of association with actin binding proteins in the cell cytosol [40]. We raised antiserum in rabbits and mice to the homologous epitope of *P. falciparum* actin I (PFL2215w, amino acids 239–253), which is conserved across most Apicomplexa (*Plasmodium, Toxoplasma, Theileria* and *Babesia* spp.) but not outside of the apicomplexan phylum. Of note, this sequence diverges at three residues from mammalian beta-actin (Fig. 1A,B). We recently reported that rabbit serum against this peptide, which we refer to as anti-Act_239–253_, reacted specifically with cell lysate from *P. falciparum* asexual stages, but showed poor reactivity with erythrocyte actin (reported in [41]). Immunoblots with rabbit and mouse antisera confirmed the specificity of this reactivity against human parasite lysate, and extended the observation to lysates of mouse malaria parasites and *T. gondii* (recognising a specific product of ∼40 kD consistent with the predicted masses of the respective actins: 41.8, 41.9 and 41.7 kD (Fig. 1C). When compared to conventional vertebrate actin antibodies the antiserum showed minimal cross-reactivity with mouse erythrocyte, human erythrocyte or human fibroblast actin (Fig. 1D). Thus, based on only a few divergent residues, an antibody that differentiates between human and parasite actin has been generated.

### Actin dynamics localise to pellicular and apical regions of motile or invasive zoites

**Figure 2:**
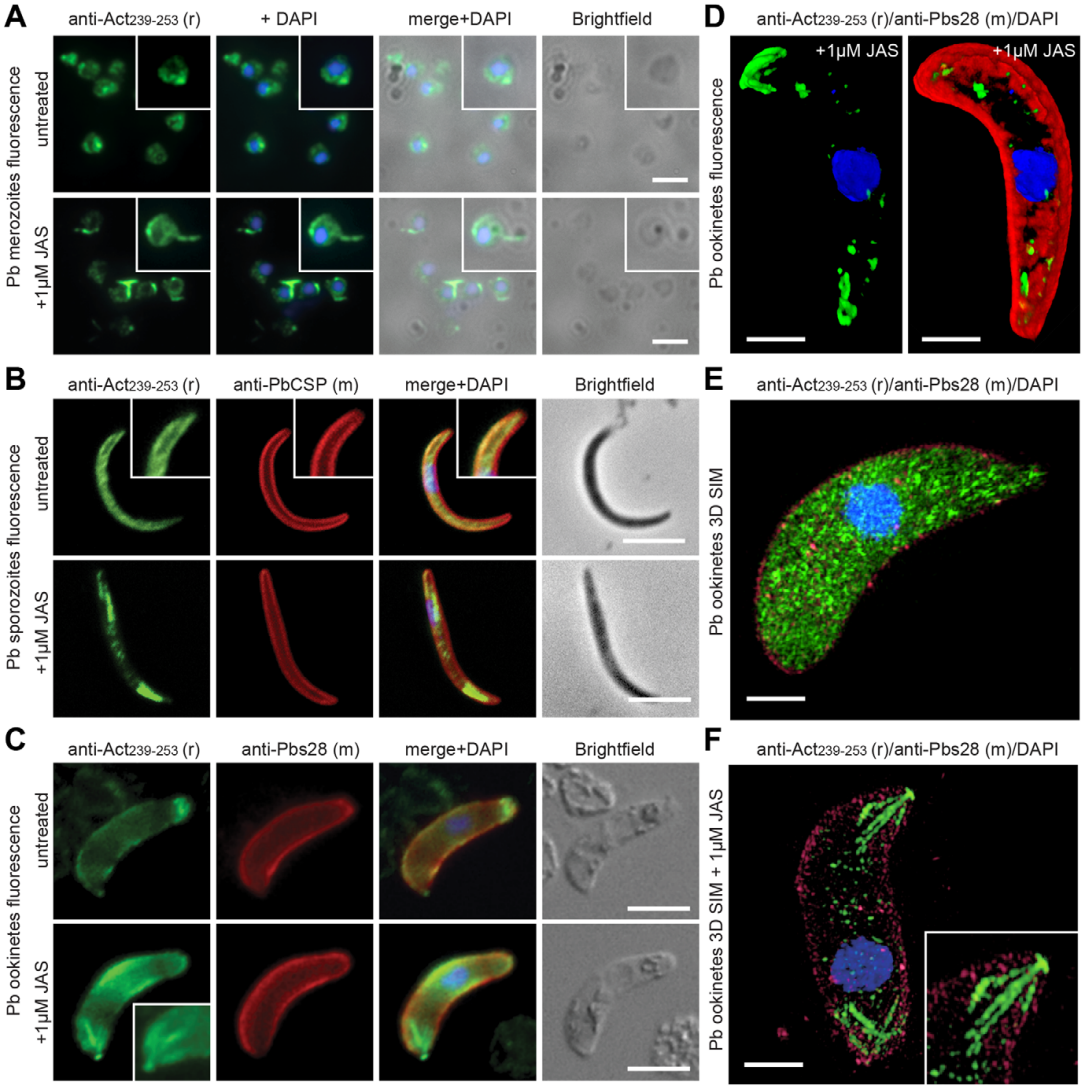
Spatial distribution of actin in free malaria parasite zoites. **A**) Widefield IFA of *P. berghei* merozoites with and without 1 µM JAS, labelled with rabbit anti-Act_239–253_ (Green) and the nuclear marker DAPI. Scale bar = 2 µm. **B**) Widefield IFA of *P. berghei* sporozoites with and without 1 µM JAS, labelled with rabbit anti-Act_239–253_ (Green), surface marker PbCSP (Red) and DAPI (Blue). Scale bar = 5 µm. See also Movie S1 **C**) Widefield IFA of *P. berghei* ookinetes with and without 1 µM JAS, labelled with rabbit anti-Act_239–253_ (Green), surface marker Pbs28 (Red) and DAPI (Blue). Scale bar = 5 µm. **D**) 3D reconstruction of widefield IFA with deconvolution of 1 µM JAS treated *P. berghei* ookinete, labelled with rabbit anti-Act_239–253_ (Green), Pbs28 (Red), and DAPI (Blue). Scale bar = 5 µm. See also Movie S2. **E–F**) 3D structured illumination microscopy (3D SIM) of *P. berghei* ookinetes labelled with rabbit anti-Act_239–253_ (Green), surface marker Pbs28 (Red) and DAPI (Blue) in the absence (**E**) and presence of 1 µM JAS (**F**). Scale bar = 2 µm. See also Movie S3, S4. Gamma settings were altered in 3D reconstructions.

To explore the general utility of anti-Act_239–253_ against all motile or invasive lifecycle stages and beyond *P. falciparum* [41], [42], we used the mouse malaria parasite *P. berghei*, which greatly facilitates generation of each zoite form: merozoite, ookinete and sporozoite. Actin labelling by immunofluorescence was seen to concentrate broadly at the pellicular regions of free merozoites, ookinetes and salivary gland sporozoites (Fig. 2A–C, upper panels). Pellicular labelling in merozoites was further supported by serial section immunoelectron microscopy of free *P. falciparum* merozoites (Fig. S1A). When treated with the cyclodepsipeptide Jasplakinolide (JAS), which arrests actin filament turnover [25], labelling became even more pronounced in pellicular and apical regions highlighting these areas as foci for actin turnover (Fig. 2A–C, lower panels). Prominent structures, reminiscent of an acrosomal process described in JAS treated *T. gondii* tachyozites [26], [28] and *P. falciparum* merozoites [24], could be seen in all three motile or invasive stages (Fig. 2A–C, lower panels). In sporozoites, labelling was generally greatest in apical and posterior regions, known sites of substrate attachment in these cells [15] (Fig. 1B, Movie S1). In ookinetes, labelling along the flanks post-JAS treatment remained associated with the pellicular region and was frequently strongest at the flexed portion of the parasite cell (Fig. 2C), which may represent a key point of traction in line with recent observations using ookinetes that express constitutive GFP-actin [21]. In ookinetes where apical or basal concentrations of labelling were strongest, three-dimensional reconstruction of fluorescent images demonstrated clear capping of the ookinete poles (Fig. 2D, Movie S2). We explored this further by three-dimensional structured illumination microscopy (3D-SIM), a technique able to resolve structures beyond the normal resolution limits of conventional fluorescence microscopy [43]. Untreated ookinetes demonstrated broad cytosolic localisation of actin with variable pellicular concentrations under 3D SIM conditions (Fig. 2E, Movie S3). Following JAS treatment, actin labelling again redistributed to polar regions with capping structures resolved into branched rod-like fibres, frequently with three or more arms (Fig. 2F, Movie S4) [21], [27]. Why these actin-rich structures should be so uniform is unclear, but they are similar to bundles of actin seen following JAS treatment by electron microscopy in *Toxoplasma* tachyzoites [26]. Electron microscopy of untreated versus JAS-treated ookinetes was unable to resolve directly the nature of these structures (Fig. S2A–B).

### Pellicular actin is concentrated in the supra-alveolar space and associates with membranes

**Figure 3:**
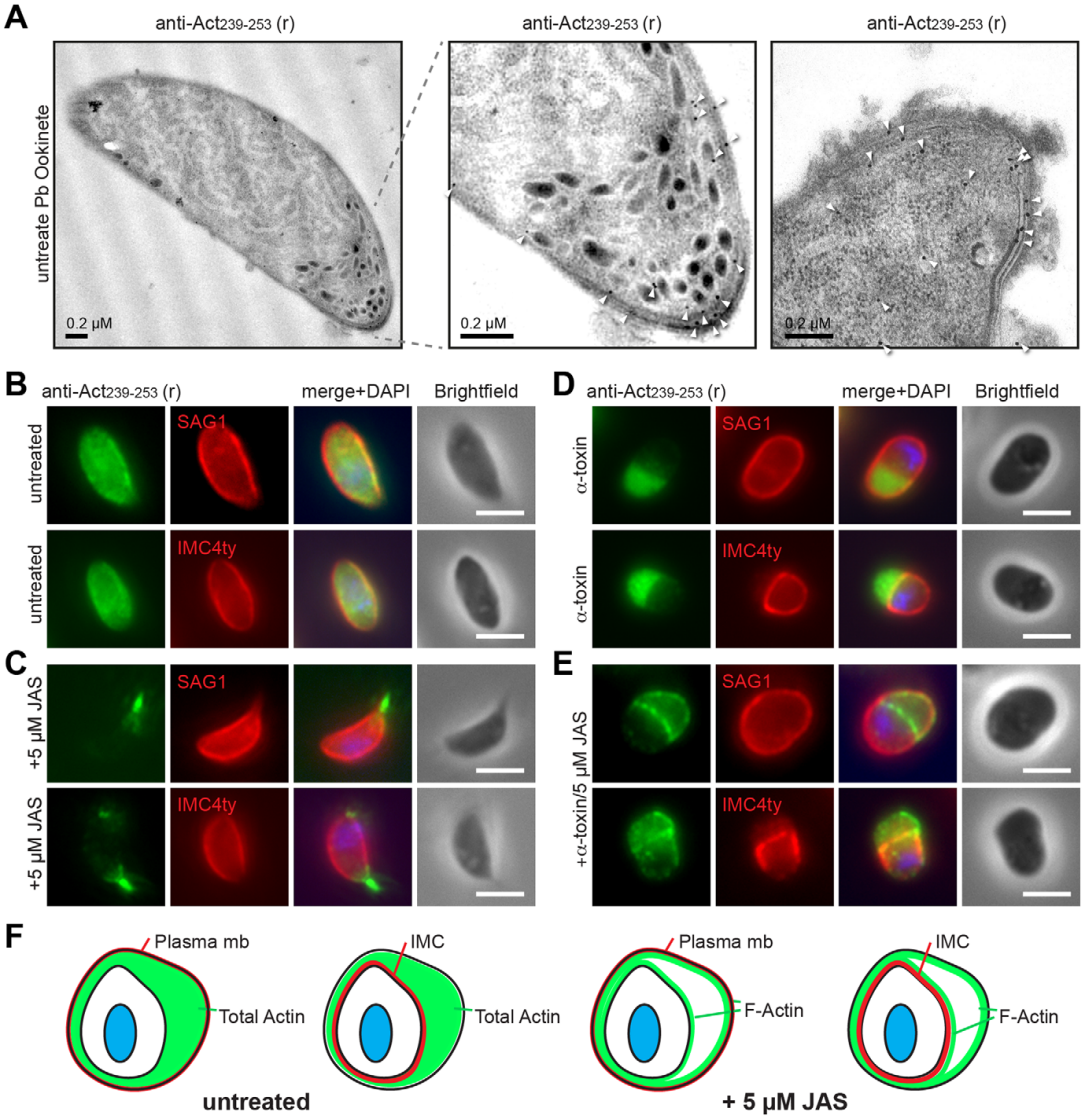
Location of anti-PfAct239–253 labelling to supra-alveolar membranes. **A**) Transmission electron micrographs with anti-Act_239–253_ (rabbit) immunogold labelling (arrowheads) of *P. berghei* ookinetes (including inset and independent ookinete apical end). **B–E**) Widefield IFA of free *T. gondii* tachyzoites under various treatments probed with anti-PfAct_239–253_ (Green) versus anti-TgSAG1 (a plasma membrane marker) or anti-Ty (IMC4, and IMC marker) (Red). DAPI (Blue) and Scale bar = 5 µm. **B**) Untreated. **C**) Following treatment with 5 µM JAS. **D**) Following treatment with *C. septicum* α toxin 1/100. **E**) Following treatment with 5 µM JAS and α-toxin. **F**) Schematic for labelling seen following treatment with α toxin alone or in combination with 5 µM JAS localising actin filaments.

Broad cortical localisation of actin in each lifecycle stage is suggestive of an association of actin with membranes in the parasite pellicle. Entirely consistent with this, carbonate extraction of *P. falciparum* schizont lysate (to separate membrane associated from cytosolic proteins) indicated a substantial portion of the actin pool does associate strongly with membranes when compared to cytosolic controls (Fig. S3A). Immunoelectron microscopy of ookinetes, labelled with anti-Act_239–253_, was consistent with a pellicular association, demonstrating strong associations of gold labelling in both apical and supra-alveolar compartments (Fig. 3A). Attempts to define conclusively the pellicular membrane associated with actin using *Clostridium septicum* alpha toxin, which has been used extensively to separate the plasma membrane away from IMC in *T. gondii* tachyzoites [9], were unsuccessful with all malaria parasite zoite forms (data not shown). Given the conservation of the reactive actin 239–253 epitope in *T. gondii* (Fig. 1A, C, Fig. S3B), we undertook immunofluorescence assays with extracellular tachyzoites treated with the pore forming toxin. Tachyzoites labelled with anti-Act_239–253_ demonstrated broad pellicular association of actin with marked redistribution following JAS treatment to apical and cortical regions (Fig. 3B–C) [28]. Using markers for the plasma membrane (SAG1) and IMC (IMC4ty, Bradin and Tonkin, unpublished data), alpha-toxin treatment defined labelling to this compartment as within the supra-alveolar space (Fig. 3D). Combining toxin treatment with JAS, labelling shifted from within the supra-alveolar space to the bounding membranes of the IMC (Fig. 3E). In conjunction with differential solubilisation (Fig. S3A) this distribution supports an affinity of the dynamic actin filament pool for the pellicular membranes that bound the supra-alveolar space (Fig. 3F).

Combined, the intensity and restriction of fluorescence labelling (following JAS treatment in particular) and lack of cross reactivity with a variety of host cells used corroborates the specificity of the anti-Act_239–253_ serum for parasite actin. Furthermore, the data provide clear support that microfilament dynamics are focussed at the apex and pellicle of motile or invasive parasite stages, likely associated with membranes of the supra-alveolar space. These observations are entirely in line with current models for actin's proposed function in apicomplexan parasites and its dominant role in driving motility through the IMC immobilised gliding motor [12].

### Actin filament dynamic also localise to the nuclear periphery in multiple lifecycle stages

**Figure 4:**
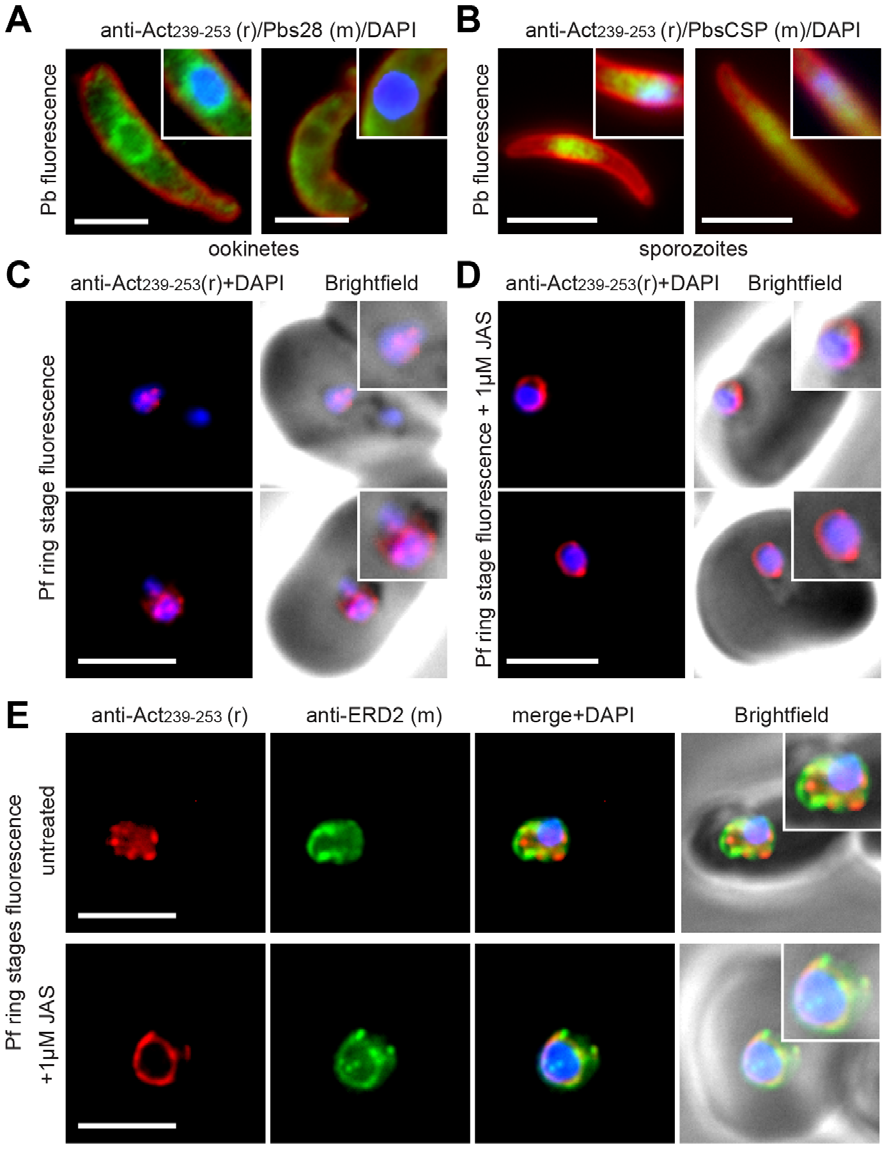
Concentration of actin labelling in the nucleus and around the nuclear periphery. Widefield IFA of representative *P. berghei* **A**) ookinetes and **B**) sporozoites that show pronounced nuclear labelling using rabbit anti-Act_239–253_ (Green) surface markers Pbs28 or PbCSP (Red) and DAPI (Blue). Scale bar = 5 µm. See also Movie S5. **C**) Widefield IFA of *P. falciparum* rings labelled with rabbit anti-Act_239–253_ (Red) and DAPI (Blue). **D**) As **C** but following 6 hour JAS treatment. **E**) Two colour widefield IFA using rabbit anti-Act_239–253_ (Red), rat anti-ERD2 (Green) and DAPI (Blue) in absence or presence of 1 µM JAS. All scale bars = 5 µm.

In several instances, immunofluorescence assay of ookinetes and sporozoites revealed a sizable proportion of actin localised at the nucleus or around the nuclear periphery (Fig. 4A, B, Movie S5). This may suggest that actin dynamics function in nuclear architecture or gene regulation, as is seen in other eukaryotes [35]. To explore the generality of this observation in other lifecycle stages (in particular those in which nuclear activity is high) early ring stage asexual parasites were labelled with anti-actin and the nuclear marker DAPI. Very early rings demonstrated a consistent punctate labelling of actin within DAPI staining of the nucleus (Fig. 4C). Treatment with JAS transformed this punctate pattern into a clear ring surrounding the nucleus (Fig. 4D). The concentration of stabilised actin filaments at the nuclear periphery was confirmed using ERD2, a cis-Golgi marker that localises to defined sites adjacent to the nucleus [44] (Fig. 4E). Demonstration of actin in the nucleus, specifically its dynamic nature at the nuclear periphery, is consistent with emerging understanding of the roles of actin and myosin in movement of chromosome ends to the nuclear periphery [45], [46]. Indeed, two recent studies have corroborated just such a role for actin in gene regulation during *P. falciparum* ring stage development [47], [48].

### The association of actin with the tight junction during merozoite and sporozoite invasion of host cells

**Figure 5:**
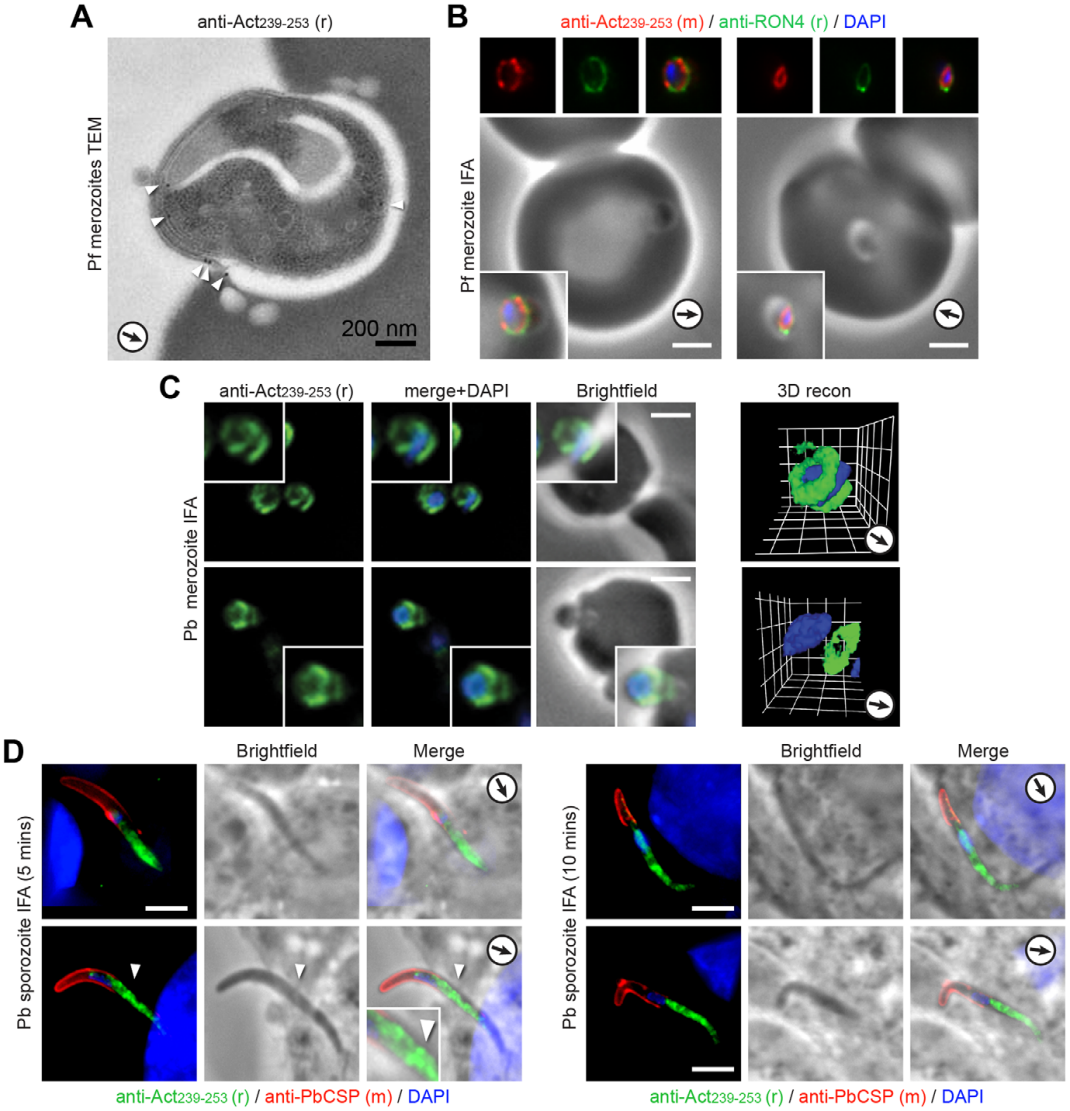
The spatial distribution of actin in invading merozoites and sporozoites. **A**) Transmission electron micrograph with anti-Act_239–253_ (rabbit) immunogold labelling (arrowheads) of invading *P. falciparum* merozoite. Arrows show direction of invasion. **B**) Widefield IFA with deconvolution of invading *P. falciparum* merozoites labelled with mouse anti-Act_239–253_ (Red) or rabbit PfRON4 (Green) and DAPI (Blue). Scale bar = 2 µm. **C**) Widefield IFA with deconvolution of invading *P. berghei* merozoites labelled with rabbit anti-Act _239–253_ (Green) and DAPI (Blue). Scale bar = 2 µm. Gamma settings were altered in 3D reconstruction. **D**) Widefield IFA with deconvolution of invading *P. berghei* sporozoites labelled with rabbit anti-Act_239–253_ (Green), anti-PbCSP (Red, exterior only) and DAPI (Blue). Scale bar = 5 µm, arrowhead shows presumed site of tight junction.

We have recently shown that rabbit serum against Act_239–253_ labels a ring of actin at the merozoite-erythrocyte tight junction during *P. falciparum* merozoite invasion [41], [42]. We confirmed the presence of a concentration of actin at the electron dense junction by immunoelectron microscopy using rabbit antiserum (Fig. 5A) and by immunofluorescence with new antiserum raised in mice (Fig. 5B). To explore the generality of this observation in other species, we undertook immunofluorescence imaging with merozoites and salivary gland sporozoites from *P. berghei*. Invading *P. berghei* merozoites showed a consistent ring of actin at the junction during invasion (Fig. 5C). Sporozoite invasion of hepatocytes is notoriously hard to capture [49]. Furthermore, sporozoites are actively involved with gliding as well as invasion (for example traversing cells in the absence of true invasion [50]) and differentiating between the two can be challenging. Sporozoites dissected from mosquito salivary glands and applied to cultured HepG2 liver cells showed a consistent, seemingly structured, concentration of actin in the internalised portion of the entering sporozoite (as marked by restriction of CSP to the exterior) (Fig. 5D, inset). In some instances patterns of labelling included a distinct band of actin associated with a tight junction constriction (Fig. 5D). However, exclusive labelling showing a ring of actin at the sporozoite junction was not seen. Whilst these data suggest that concentration of actin in a ring at the host-parasite tight junction is a clear feature of merozoite invasion across species, its conservation in other zoite forms is still uncertain, likely confounded by the presence of traversal and general gliding in addition to invasion in these forms.

### Anti-Act_239–253_ shows preferential labelling for actin filaments

**Figure 6:**
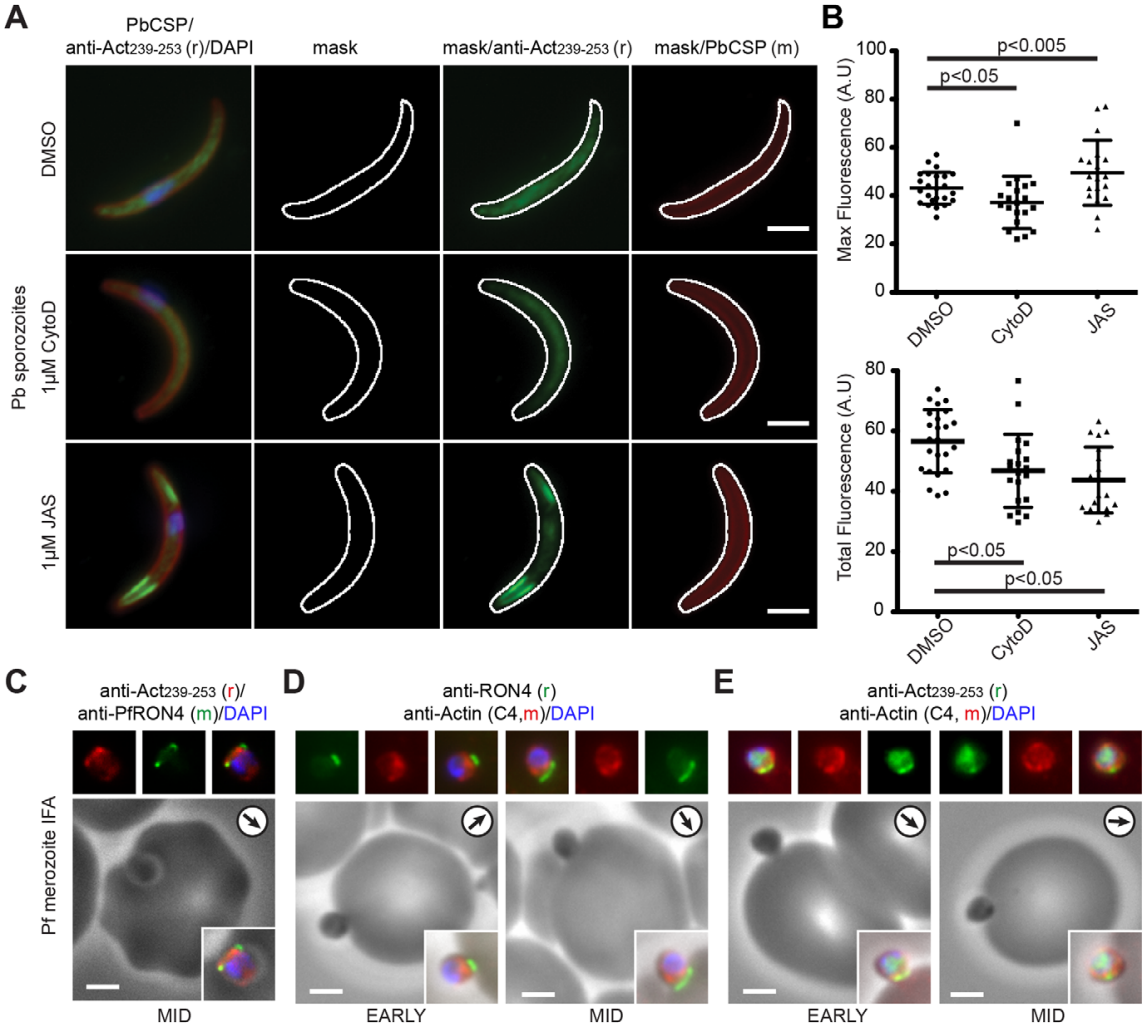
Anti-Act_239–253_ shows preferential labelling of actin filaments. **A**) Widefield IFA with deconvolution of sporozoites treated with DMSO control, 1 µM cytochalasin D or 1 µM JAS labelled with rabbit anti-Act_239–253_ (Green), PbCSP (Red) and DAPI (Blue). Scale bar = 2 µm. Mask determined by anti-PbCSP labelling (see Materials and Methods). **B**) Maximum and total fluorescence levels of sporozoites treated with 1 µM JAS, 1 µM cytochalasin D and DMSO control. Significance as shown, unpaired t-test. **C**) Widefield IFA with deconvolution of invading *P. falciparum* merozoite labelled with rabbit anti-Act_239–253_ (Red) mouse anti-PfRON4 (Green) and DAPI (Blue). Scale bar = 2 µm. Arrows show direction of invasion. **D–E**) Widefield IFA with deconvolution of invading *P. falciparum* merozoites labelled with mouse anti-Actin (C4, Red) co-labelled with rabbit anti-PfRON4 (Green) (**D**) or rabbit anti-Act_239–253_ (Green) (**E**). DAPI (Blue) and Scale bar = 2 µm.

Given the unusual reactivity of the parent anti-Gly_245_ antibody, we sought to explore the filament labelling preferences of the anti-Act_239–253_ antibody using quantitative imaging of sporozoites treated with either JAS (to stabilise filaments), cytochalasin D (in which filaments are capped and will be less prevalent) or left untreated (Fig. 6A). We reasoned that if the antibody recognises filament ends, fluorescence intensity should increase in cells that have more filaments. In agreement with this, maximum fluorescence intensity for JAS treated sporozoites (where the number of normally transient filament ends is stabilised and concentrates in areas of dynamic actin) was significantly higher (p<0.005, unpaired t-test), whereas that for cytochalasin D treated parasites (having reduced F-actin) was significantly lower (p<0.05, unpaired t-test) than untreated controls (Fig. 6B). Total fluorescence decreased for both treatments (Fig. 6B). This decrease in maximal fluorescence intensity would be expected for cytochalasin D. Following JAS-treatment, the reduction likely results from two contributing factors. First, since JAS treatment elongates filaments, the total number of filament ends may be reduced. Second, whilst sporozoites that were relatively flat in the plane of view were selected for observation, 3D reconstructions demonstrated that a significant proportion have fluorescence that frequently lies outside the plane of imaging therefore removing a major proportion of actin labelling from quantification (Fig. S4). As further evidence, we re-visited *P. falciparum* merozoite invasion, reasoning that since actin filaments are required for invasion, the tight junction is likely filamentous in nature whilst non-polymerised actin (recognised by a generic anti-Actin antibody) would be cytosolic. Fluorescent labelling with anti-Act_239–353_ was again concentrated to the junction (co-labelling with RON4) (Fig. 6C), whereas that seen when using a generic anti-Actin monoclonal (C4) showed a broad cytosolic distribution not restricted to the junction or to regions of anti-Act_239–353_ labelling (Fig. 6D, E). Combined, this evidence would support anti-Act_239–253_ having a strong affinity and potential preference for actin in a filamentous over monomeric state and the existence of actin filaments at the tight junction. By extension, this also implies that pellicular and apical actin labelling in free motile and invasive zoite forms (Fig. 2–3) and peripheral nuclear actin labelling across development stages (Fig. 4) is likely of a filamentous nature.

### Imaging of actin filament-like structures at the tight junction

**Figure 7:**
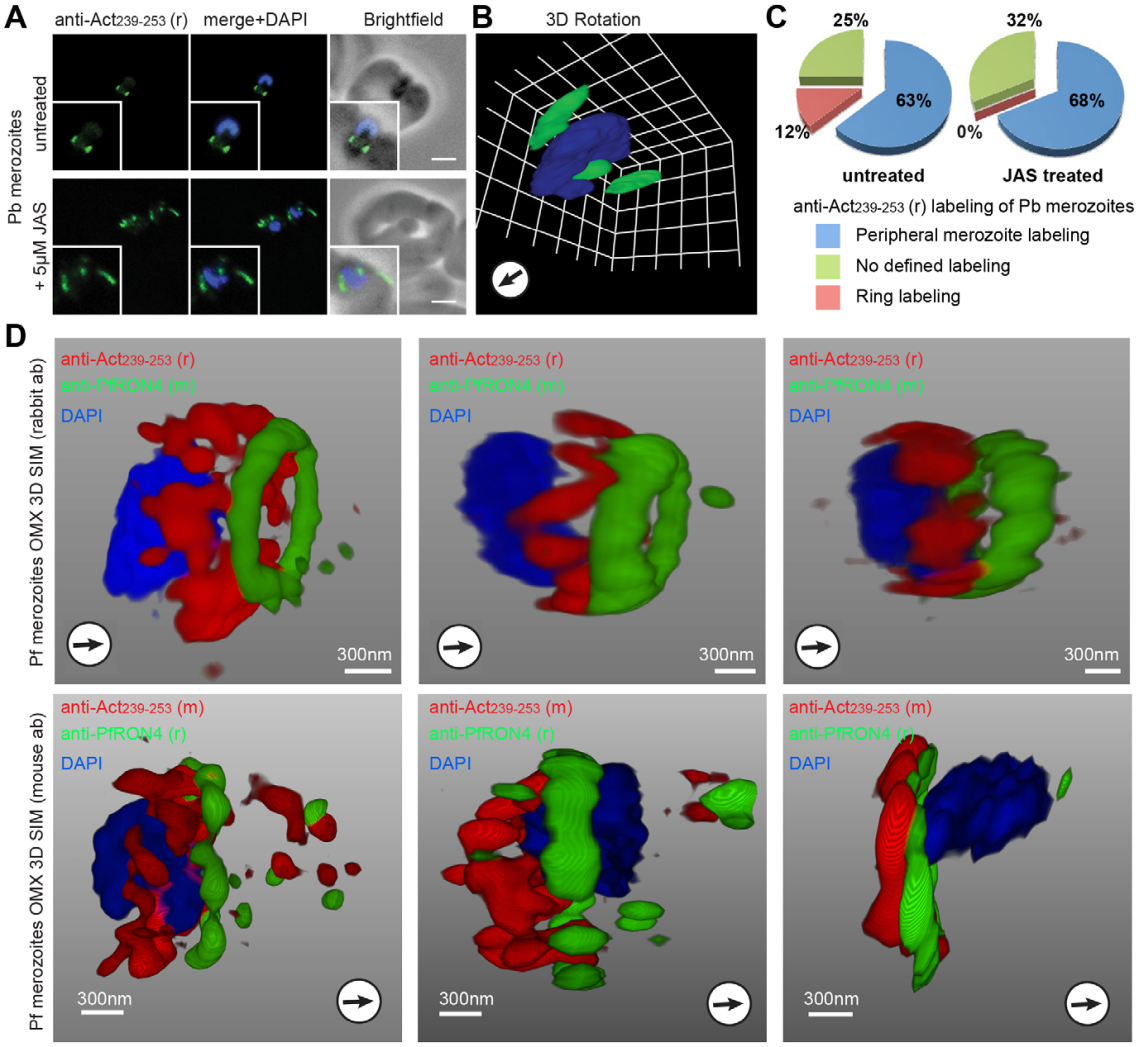
The tight junction is composed of dynamic actin filaments that localise posterior to the junction during invasion. **A**) Widefield IFA with deconvolution and **B**) 3D reconstruction of *P. berghei* merozoites incubated with and without 1 µM JAS and labelled with anti-Act_239–253_ (Green) and DAPI (Blue). Scale bar = 2 µm. Arrows show direction of invasion. **C**) Graphic representation of actin labelling in *P. berghei* merozoites with and without the addition of JAS. *n* = 124 merozoites for each of three replicates, mean is shown. **D**) 3D structured illumination microscopy (3D SIM) of three separate invading *P. falciparum* merozoites labelled with rabbit (upper row) and mouse (lower row) anti-Act_239–253_. Labelling shows actin (Red), RON4 (Green) and DAPI (Blue). See also Movie S6. Gamma settings were altered in 3D reconstructions.

It has previously been shown that treatment of merozoites with actin inhibitors arrests invasion subsequent to tight junction formation [23], [41]. To further explore the nature of actin at the tight junction, we compared untreated merozoites with those treated with high concentrations of JAS (post-attachment) to prevent complete invasion (see Materials and Methods). When compared to untreated controls (Fig. 7A, upper panel), *P. berghei* merozoites incubated with erythrocytes but treated with JAS following attachment showed a breakdown of anti-Act_239–253_ labelling (Fig. 7A, lower panel). Instead of a clear ring of actin, labelling appeared in elongated furrows surrounding the invading parasite (Fig. 7B). Similar results were seen with *P. falciparum* merozoites (*data not shown*). Given the greater numbers of invading merozoites seen with *P. berghei*, we attempted quantification of actin labelling under different treatments. Whilst circumferential (i.e. pellicular) actin labelling of merozoites associated with erythrocytes (as seen in free merozoites in Fig. 2A) was found to be similar for both treatments, no actin ring labelling was seen for any merozoite following JAS treatment (Fig. 7C). These data demonstrate that the ring like formation of actin at the tight junction, and its maintenance through invasion [41], is not a static structure but instead is dependent on filament turnover.

To resolve the architecture of the actin ring at the junction further, *P. falciparum* merozoites labelled with both rabbit (r) and mouse (m) anti-Act_239–253_ serum were imaged using three-dimensional 3D-SIM, an approach that we have recently used to provide insight into the structures formed during *P. falciparum* erythrocyte infection [41]. In all instances Anti-Act_239–253_ labelling was concentrated in a ring lying posterior to the tight junction during merozoite invasion, defined as the edge of the junction towards the posterior of the parasite (Fig. 7D, Movie S6). However, labelling was rarely seen overlapping with the junction plane. Furthermore, on occasion the distribution of fluorescent signal clearly showed short filament-like structures around the circumference of the tight junction, with each fibre running approximately parallel to the plane of merozoite invasion. Although accurate sizing of these filaments is beyond the resolution limits of 3D-SIM, their size (less than 500 nm, Fig. 7D, Movie S6) is entirely consistent with the *in vitro* determined length of actin filaments from apicomplexan parasites [31], [32], [33]. These images may represent the first time that actin filaments (or bundles thereof) have been seen under conventional and drug free imaging conditions in motile apicomplexan parasites. Combined with the results following JAS treatment, these data suggest dynamic actin filaments are a critical component of the tight junction. However, contrary to expectations, filaments reside behind and not directly in the plane of the junction, in contrast with labelling of apical membrane antigen (AMA) 1 [41]. This suggests that the driving force for motility (via actin-myosin) and the architecture of the tight junction (via AMA1-RON complex interaction) are discrete entities during invasion.

## Discussion

Understanding of the role of actin in malaria parasite development has focussed in the most part on its core function during motility, though other auxiliary roles in development are being increasingly explored [33], [34], [47], [48]. The current accepted model for apicomplexan motility and the role actin plays in cell movement [8] draws much of its support from immunoprecipitation of core components of the gliding motor and associated proteins [8], [9], [11], [13], [51], [52] with some topological support from microscopic studies [8], [9], [11], [13], [18], [28], [53]. Although actin filaments form an essential dynamic component of the active parasite motor [5], [15], [22], [23], [24], [26], [28], [54], [55], [56], much of the evidence so far has been based on experiments that lack the ability to dissect, on a fine scale, precisely where the microfilaments localise in the cell. This is also true for studies exploring the role of actin in hemoglobin uptake [33] and vesicle trafficking [34]. Until now, the definitive localisation of filaments under native imaging conditions has not been possible [19], [20], [28]. Here, utilising a parasite specific actin antibody that demonstrates preferential labelling of actin filaments, we show that F-actin is directly associated with several key compartments of the parasite indicative of separate functions. These include the nuclear periphery and F-actin association with gene regulation, pellicular membranes of zoite forms and cell motility, and the host-parasite tight junction and the essential role played by actin in merozoite invasion.

Nuclear localisation of dynamic actin is consistent with recent studies that have demonstrated a role for actin and myosin in the movement of chromosome ends to the nuclear periphery in human cells [45], [46]. Movement of active genes to sub-compartments within the nucleus has been shown to function in regulating antigenic variation in blood stage malaria parasites [57], [58]. Indeed, two recent studies have demonstrated a key role of actin in both the spatial repositioning of genes in the nucleus and in binding to a regulatory nuclear histone methyltransferase [47], [48]. As such, dynamic actin is clearly a key factor involved in mediating parasite antigenic variation and gene activation. Further investigation of the parasite nucleus in high definition and the effects of actin inhibitors on gene regulation will likely prove significant areas of interest for probing this possible function.

Association of actin filaments with the supra-alveolar space and its association with pellicular membranes is clearly in line with the current model for gliding motility. At present, however, it is not clear which, if either, pellicular membrane provides the native binding surface. A compelling model would be for filaments of actin to directly associate with the plasma membrane. In other systems membrane bound glycosyl-phosphatidylinositol anchored proteins play a key role in linking F-actin directly to cortical membranes [59]. If validated in an apicomplexan cell, such a scenario would negate the need for a linear relationship linking actin filaments to the surface via tetrameric aldolase and the cytoplasmic tails of secreted thrombospondin related anonymous protein (TRAP)-family adhesins [11]. Of note, two-dimensional rafts of actin filaments associated with positively charged lipid layers form an ordered array when mixed with aldolase *in vitro* [60], [61]. Indeed, were such an organisation to exist underlying the plasma membrane (as might be suggested by EM data [20], [28]), this would directly provide the key rigid element upon which myosin bears its traction, transferring the entire plasma membrane raft rewards. External adhesins/invasins that cluster to this raft, perhaps anchored through interactions with aldolase [11], [13], would then also be drawn rearwards. This might also imply that no singular adhesin/invasin-host receptor interaction necessarily mediates motility but instead it is the movement of the entire cluster of proteins through the membrane, and its interaction with the external milieu that moves an apicomplexan cell. The behaviour of sporozoite adhesion foci during motility would certainly favour the existence of raft- or patch-like traction [15]. Such a raft-like model linking the IMC bound motor with external adhesins is consistent with current data, and would only challenge the terminal link between actin and the extra-cellular milieu [11].

**Figure 8:**
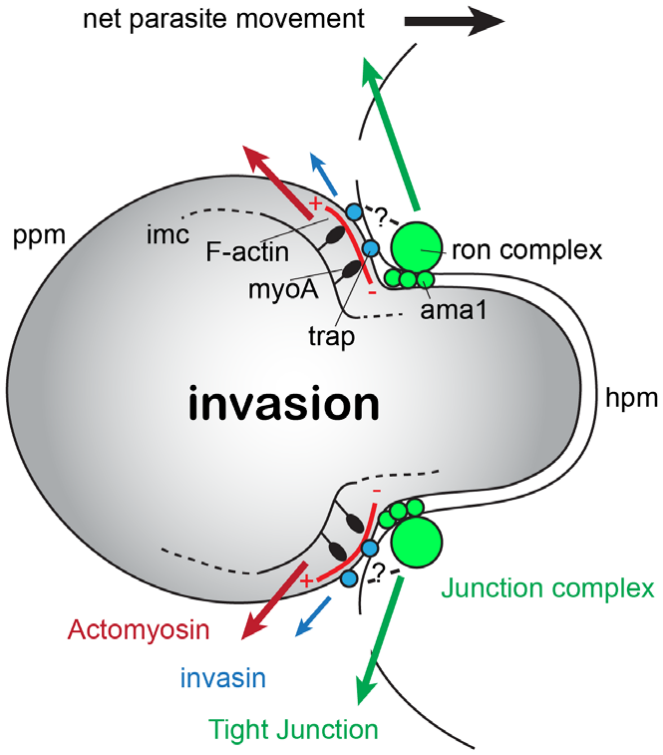
A refined model for the apicomplexan host-cell invasion. An apicomplexan zoite during host-cell invasion. Abbreviations: ama1, apical membrane antigen 1; F-actin, actin filaments (turning over); hpm, host plasma membrane; imc, inner membrane complex; myoA, myosin A; ppm, parasite plasma membrane; ron-complex, rhoptry neck protein complex; trap, thrombospondin related anonymous protein. Question mark indicates unknown linkage between motor complex (actin and myosin) and tight junction complex (ama1-ron complex). Red arrow indicates movement of actin filaments rearwards by myoA driving motility. Movement of actin drives trap-like adhesin (Blue arrow). Green arrow marks movement of traction point complexes (light green) during gliding or invasion.

An important refinement to our current model of gliding mechanics during invasion may be required in light of the placement of actin filaments behind the tight junction and not directly beneath it. Recent evidence for the *in vitro* interaction of AMA1 with aldolase has been used to argue that AMA1 may interact directly with the gliding motor, leading to the notion that force generation and attachment are one contiguous structure [62], [63]. Our imaging evidence supports an alternative model where force from the actomyosin motor is transmitted indirectly to the tight junction [64] (Fig. 8). If verified, such a model would raise several key questions. First, how is actin polymerisation spatially restricted to such a localised site? Since apicomplexan parasites possess a markedly reduced repertoire of identifiable actin regulators [12] this dramatically shortens the list of potential actin-binding proteins that might govern filament spatiotemporal localisation. A likely factor may be Formin1, which in malaria parasites has been tentatively localised to the tight junction [38]. However, this does not entirely agree with recent work in *T. gondii*, which demonstrates that of the two conserved formins (Formin1 and 2) both localise to the pellicle of the motile or invasive zoite but not specifically to the junction during invasion [65]. Discrepancies between the focussed ring of actin seen during merozoite invasion but not sporozoites invasion (Fig. 5) (nor that of tachyzoites, *data not shown*) may reconcile these conflicting observations. Recently, we have demonstrated that *P. falciparum* actin depolymerising factor 1 (ADF1) has a broad cytosolic localisation but is excluded from the tight junction during merozoite invasion [42]. As such it may be a combination of nucleation, stabilisation and depolymerisation factors that ultimately dictates the restricted localisation of actin to the tight junction zone. A second question is what links the actin (and possibly aldolase) raft along with its associated surface bound adhesins to the tight junction RON-AMA1 complex? Proteins responsible for this linkage would be of great interest.

Ultimately, precise imaging of each individual component of the motility and invasion machinery by fixed and live imaging approaches is required to resolve current models for the molecular basis of gliding. Live imaging of dynamic actin in particular, and the development of tools for this purpose, would be a major step towards this goal. Furthermore, increased efforts in understanding the regulation of actin's spatiotemporal localisation, filament turnover, and association with the plasma membrane may help explain how motility is regulated. More broadly, efforts to understand the repertoire of functions played by actin in malaria parasite development and movement should reveal potent chemotherapeutic targets that transcend stage specificity and may work as the basis for treatment and also transmission blocking drugs.

## Materials and Methods

### Ethics statement

The culture of *P. falciparum* parasites using donated blood and serum from the Australian Red Cross Society and use of mice for growing *P. berghei* have been approved by The Walter and Eliza Hall Institute Human Ethics (HEC 86/17) and Animal Ethics Committees (AEC Project 2009-023).

### Antibody production

A synthetic peptide spanning amino acids 239–253 of PfActin (PlasmoDB ID: PFL2215w) was used to raise polyclonal rabbit antisera (Genscript, USA) and mouse antisera against malaria actin (anti-Act_239–253_).

### Parasite culture and maintenance and immunoblot analysis

*P. falciparum* (3D7 and D10 strains), *P. berghei* (ANKA strain) and *T. gondii* (RH strain) parasites were each maintained using standard procedures. *P. falciparum* cultures were grown in human O+erythrocytes at 4% hematocrit with 0.5% Albumax II (Invitrogen). 3D7 is a cloned line derived from NF54, obtained from the late David Walliker at Edinburgh University, UK. *P. berghei* lines were maintained in Balb/c mice as described previously [66]. *In vitro* conversion to ookinetes followed Moon *et al.* [67]. *T. gondii* was propagated in human foreskin fibroblasts (HFF) grown in Dulbecco's modified Eagle's medium (DMEM) with 1% fetal calf serum (GIBCOBRL). For α-toxin treatment, needle passaged IMC4-ty transfectant tachyzoites (C. H. Bradin and C. J. Tonkin, unpublished) were incubated in 50 µl of *Clostridium septicum* culture supernatant. Lysates from saponin-treated schizont stage *in vitro* cultures of both *P. falciparum* and *P. berghei* along with tachyzoites were harvested by centrifugation, resuspended in reduced sample buffer and analysed by Western blot probing with rabbit or mouse anti-Act_239–253_ antisera at 1∶1000 and 1∶200, with anti-Actin (clone C4, Millipore) at 1∶1000. Signal was detected by anti-rabbit or mouse IgG horseradish peroxidase conjugate (HRP) (Millipore), and visualised via enhanced chemiluminescence (ECL, Amersham Biosciences). For solubility analysis, isolated 3D7 strain *P. falciparum* merozoites resuspended in water (Complete Protease Inhibitors, Roche) were snap frozen and incubated on ice for 10 min to release the cell content. Water soluble and insoluble proteins were separated by ultracentrifugation at 100,000× g for 30 min at 4°C (TLA100.2 rotor, Beckman Optima TL Ultracentrifuge, Beckman Coulter). Water insoluble fractions were further treated with Na_2_CO_3_ pH 11.5 for 1 hour at 4°C. Carbonate soluble and insoluble fractions were isolated by further ultracentrifugation. Samples were subject to SDS-PAGE and immunoblot analysis. Membranes were incubated with antisera (rabbit anti-Act_239–253_ [1∶500], rabbit anti-PfADF1 [1∶1000] [42] and rabbit anti-MTRAP [1∶200] [13]).

### Zoite invasion preparation

Blood stage *P. falciparum* parasites and *P. berghei* (ANKA) were cultured through to schizogony and prepared for merozoite invasion following Boyle et al [68]. For sporozoite invasion *P. berghei* sporozoites were dissected from infected *Anopheles stephensi* salivary glands and kept on ice in HepG2 culture media (Advanced MEM (Gibco®) supplemented with 10% foetal calf serum (Bovogen), 1% L-Glutamin (Thermo Scientific), 1% Penicillin/Streptomycin (Thermo Scientific), 0.1% Amphotericin B (Thermo Scientific) until dissection was completed. Sporozoites were added to 1×10^5^ HepG2 cells per well in a 24 well plate. The plate was spun at 800 rpm at 4°C for 4 min to facilitate sporozoite – HepG2 cell interaction. The plate was put at 37°C and 5% CO_2_ for 5 or 10 min, carefully washed once with PBS and invasion stopped by fixation with 4% paraformaldehyde. Labelling with antibodies against PbCSP (see below) preceded Triton X-100 permiabilisation [69] to identify sporozoites mid way through invasion, followed by re-fixation and permiabilisation for intra-cellular actin labelling. *T. gondii* tachyzoite invasion was captured using a potassium shift protocol (Kafsack et al., 2004). For JAS treatment of invasion, filtered *P. falciparum* and *P. berghei* merozoites were allowed to invade erythrocytes shaking at 37°C for 1.5 and 2 min respectively before treatment with 5 µM JAS then returned to invasion assay conditions for a further 1.5 or 2 min.

### Indirect immunofluorescence assay (IFA), electron microscopy and 3D SIM imaging

#### Immunofluorescence microscopy (IFA)

*P. falciparum* merozoites, *P. berghei* merozoites or sporozoites allowed to invade were fixed in solution and prepared for IFA as described [69] using 0.0075% glutaraldehyde/4% paraformaldehyde (ProSciTech, Australia) in PBS for merozoites, 4% paraformaldehyde in PBS for sporozoites, or (for ring stages) by cold methanol fixation [13]. For JAS treatment of rings *P. falciparum* late stage cultures were allowed to invade erythrocytes before commencing treatment with 1 µM JAS for 6 hours prior to imaging. Primary antisera in 3% BSA/PBS included rabbit anti-Act_239–253_ [1∶300]; mouse anti-Act_239–253_ [1∶100]; mouse anti-PfRON4 [70]; mouse anti-PbCSP [1∶5000] [71]; mouse anti-Pb28 [72] [1∶10,000]; rat anti-ERD2 [1∶200] (MR4, ATCC Manassas Virginia); mouse anti-TgSAG1 (DG52) (Morisaki et al., 1995) [1∶5,000] (a kind gift from L. D. Sibley, Washington University School of Medicine, USA); and mouse anti-Ty (Bastin et al., 1996) [1∶1000]. Following washes appropriate secondary antibodies (Alexa Fluor-488, 594, Invitrogen) were at 1∶500 before mounting in VectaShield® (Vector Laboratories) with 0.1 ng/µL 4′,6-diamidino-2-phenylindole, DAPI (Invitrogen). Fluorescence images were obtained using a Plan-Apochromat 100×/1.40 oil immersion Phase contrast lens (Zeiss) on an AxioVert 200 M microscope (Zeiss) equipped with an AxioCam Mrm camera (Zeiss). Z-stacks were taken well above and below parasites and processed using the Axiovision release 4.7 or 4.8 deconvolution software package.

#### Ultrastructural electron microscopy

*P. berghei* ookinetes (untreated or 1 µM JAS treated) were fixed in suspension with a freshly prepared solution of 2.5% glutaraldehyde in 0.1 M phosphate buffer (PB) (pH 7.4) for 1 hr on ice. These were washed (×3) and fixed in 1% osmium tetroxide (ProSciTech, Australia) in 0.1 M phosphate buffer for 1 hr. Following extensive rinsing in distilled water samples were stained with 2% uranyl acetate (SPI-Chem, Australia) in water for 1 hr. Washed samples were dehydrated with ethanol and embedded in LR Gold Resin (ProSciTech,Australia). Following polymerisation by benzoyl peroxide (SPI-Chem, USA) ultrathin sections (80–90 nm) were cut on a Leica Ultracut R ultramicrotome (Wetzlar). After double contrasting with uranyl acetate and lead citrate sections were examined at 120 kV on a Philips CM120 BioTWIN Transmission Electron Microscope.

#### Immunoelectron microscopy

Free or invading *P. falciparum* merozoites [41] or *P. berghei* ookines were fixed in 1% glutaraldehyde (ProSciTech, Australia) on ice for 30 min. Samples were pelleted in low-melt agarose before being transferred into water. Dehydration and sectioning was as above. For labelling, sections were blocked in PBS containing 0.8% (wt/vol) bovine serum albumin and 0.01% (wt/vol) Tween 80 and then incubated in anti-Act_239–253_ diluted in the above-mentioned solution. Samples were washed and incubated with secondary antibodies conjugated to 10 nm diameter gold particles (BioCell). Post-staining with 2% aqueous uranyl-acetate and 5% triple lead prior to imaging.

#### 3D structured illumination microscopy (3D SIM)

Samples were prepared as for IFA mounted in VectaShield® (Vector Laboratories). Imaging was performed using a DeltaVision OMX 3D Structured Illumination Microscopy System® (OMX 3D-SIM, Applied Precision Inc, Issaquah, USA) as described [41].

### Image processing and actin quantification

Deconvolved Z-stacks were reconstructed in 3D, with interpolation, using Imaris version 7.1.0 (Bitplane Scientific). For clarity of display, gamma settings were altered on 3D reconstructions after deconvolution, however no comparisons of labelling levels were made from such altered images. Maximum and total actin fluorescence calculations were performed in Metamorph version 7.7.0 (Molecular Devices), within masked regions determined using the “auto threshold for light objects” function on the CSP labelling channel of sporozoites. Statistics were calculated using a student's t-test in Prism version 5 (GraphPad). General image handling was undertaken using either Image J or Adobe Photoshop CS4. Final images were assembled in Adobe Illustrator CS4 for figure generation.

## Supporting Information

Figure S1 Serial section, transmission electron micrographs with anti-Act_239–253_ (rabbit) immunogold labelling (arrowheads) of free *P. falciparum* merozoite. Scale bar = 0.2 µm.
(TIF)

Figure S2 Transmission electron micrographs of untreated and 1 µM JAS treated *P. berghei* ookinetes. Scale bar = 0.5 µm.
(TIF)

Figure S3 **A**) Western blot of *P. falciparum* schizont lysate fractionated by hypotonic lysis with subsequent carbonate extraction: labelling with anti-MTRAP (a membrane bound control) and PfADF1 (a cytosolic control). P = pellet fraction; S = supernatant fraction. **B**) Widefield IFA of intracellular *T. gondii* tachyzoites within HFF cells labelled with rabbit anti-Act_239–253_. Scale bar = 5 µm.
(TIF)

Figure S4 3D reconstruction of widefield IFA with deconvolution of independent sporozoites treated with 1 µM JAS and labelled with PbCSP (Red), rabbit anti-Act_239–252_ (Green) and DAPI (Blue). Grid = 1 µm. Asterisk marks parasite apex. See also Movie S1. Gamma settings were altered in 3D reconstructions.
(TIF)

Movie S1 Rotation of immunofluorescence imaging of 1 µM JAS treated *P. berghei* sporozoite, labelled with anti-PbCSP (Red), rabbit anti-Act_239–253_ (Green) and DAPI (Blue). See Figure 2.
(MOV)

Movie S2 Rotation of immunofluorescence imaging of 1 µM JAS treated *P. berghei* ookinete, labelled with Pbs28 (Red), rabbit anti-Act_239–253_ (Green) and DAPI (Blue). See Figure 2.
(MOV)

Movie S3 Rotation of 3D-SIM imaging of *P. berghei* ookinete, labelled with Pbs28 (Red), rabbit anti-Act_239–253_ (Green) and DAPI (Blue). See Figure 2.
(MOV)

Movie S4 Rotation of 3D-SIM imaging of *P. berghei* ookinete following 1 µM JAS treatment, labelled with Pbs28 (Red), rabbit anti-Act_239–253_ (Green) and DAPI (Blue). See Figure 2.
(MOV)

Movie S5 Rotation of immunofluorescence imaging of *P. berghei* ookinete, labelled with Pbs28 (Red), rabbit anti-Act_239–253_ (Green) and DAPI (Blue) highlighting labelling at nuclear periphery and pellicle. See Figure 3.
(MOV)

Movie S6 Rotation of 3D-SIM imaging of *P. falciparum* merozoite invading, labelled with RON4 (Green), rabbit anti-Act_239–253_ (Red) and DAPI (Blue). See Figure 6.
(MOV)
