# Supplementary figures and images for: Spatial Localisation of Actin Filaments across Developmental Stages of the Malaria Parasite
Source: PLoS One. 2012 Feb 28;7(2):e32188. doi: 10.1371/journal.pone.0032188 (PMC3289632; doi:10.1371/journal.pone.0032188)

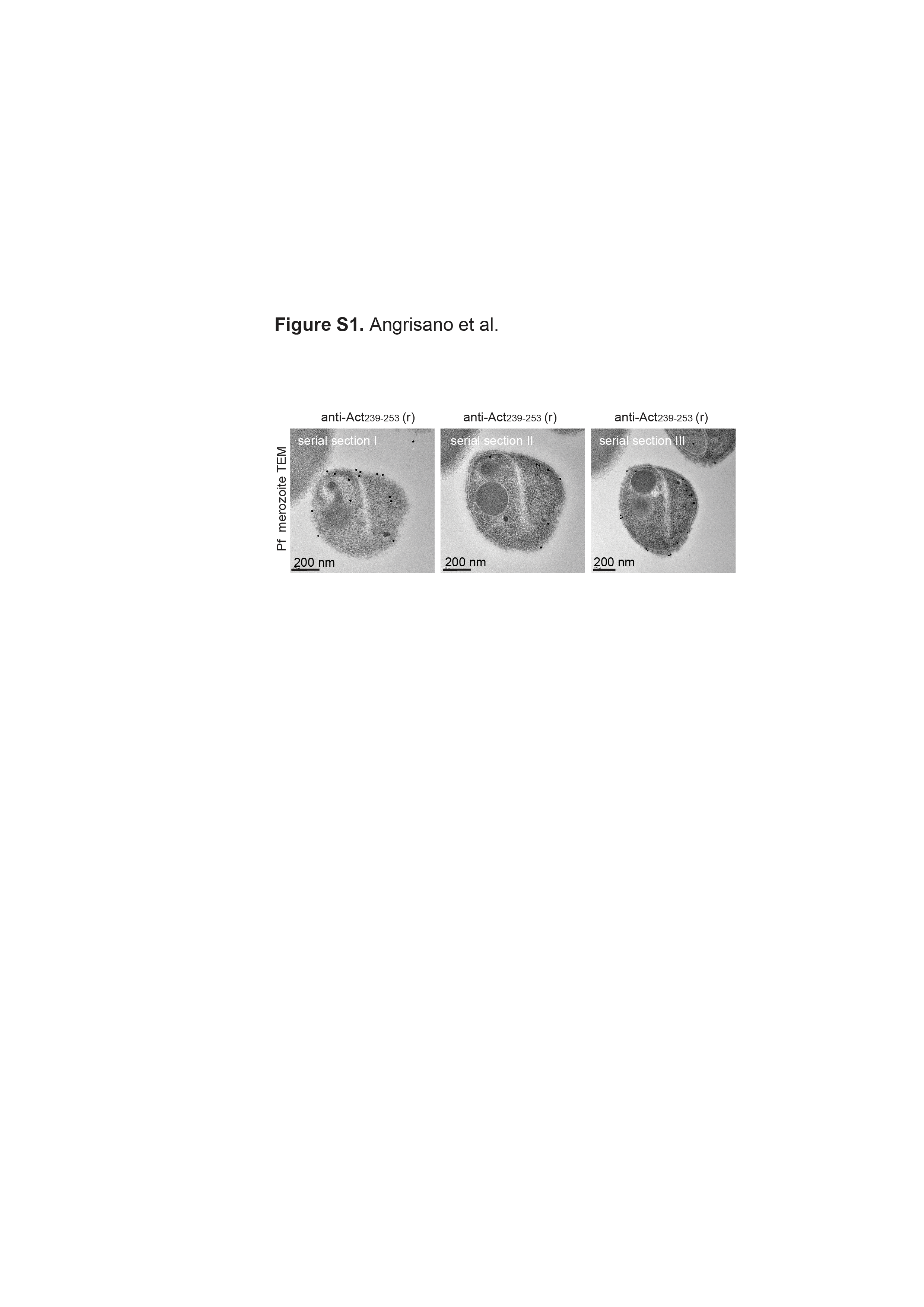

Supplement: Figure S1 — Serial section, transmission electron micrographs with anti-Act239–253 (rabbit) immunogold labelling (arrowheads) of free P. falciparum merozoite. Scale bar = 0.2 µm. (TIF) [file pone.0032188.s001.tif]

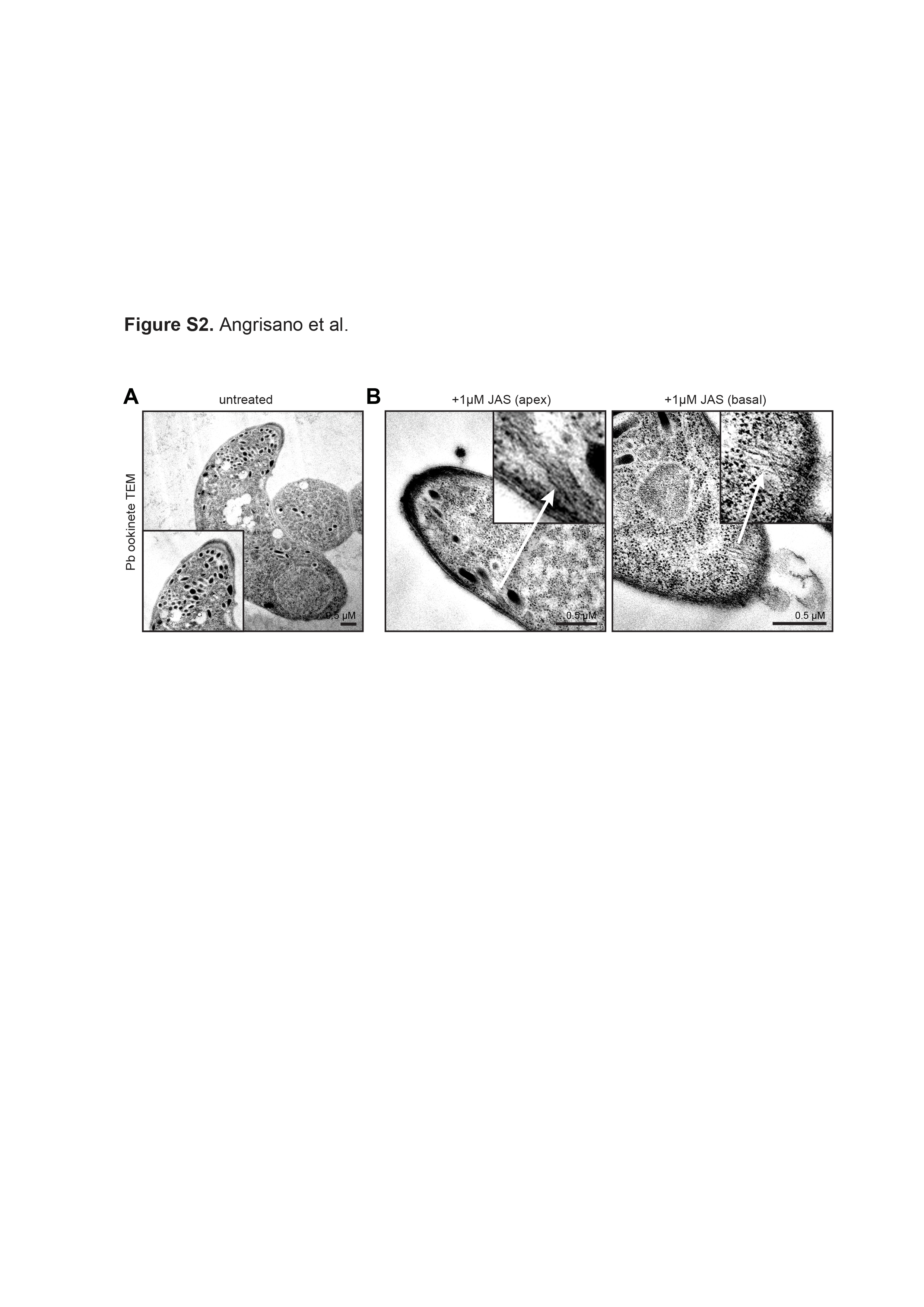

Supplement: Figure S2 — Transmission electron micrographs of untreated and 1 µM JAS treated P. berghei ookinetes. Scale bar = 0.5 µm. (TIF) [file pone.0032188.s002.tif]

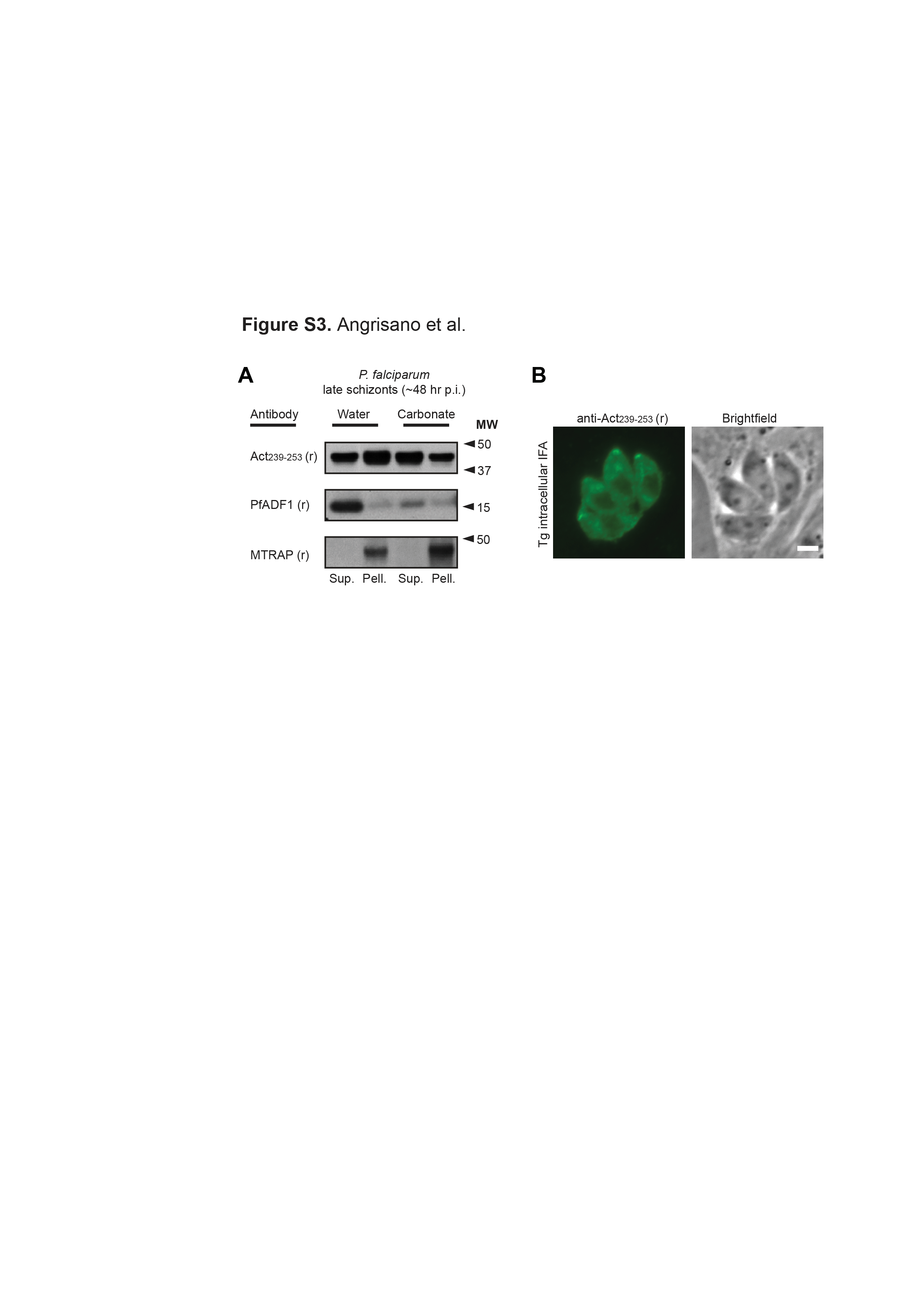

Supplement: Figure S3 — A) Western blot of P. falciparum schizont lysate fractionated by hypotonic lysis with subsequent carbonate extraction: labelling with anti-MTRAP (a membrane bound control) and PfADF1 (a cytosolic control). P = pellet fraction; S = supernatant fraction. B) Widefield IFA of intracellular T. gondii tachyzoites within HFF cells labelled with rabbit anti-Act239–253. Scale bar = 5 µm. (TIF) [file pone.0032188.s003.tif]

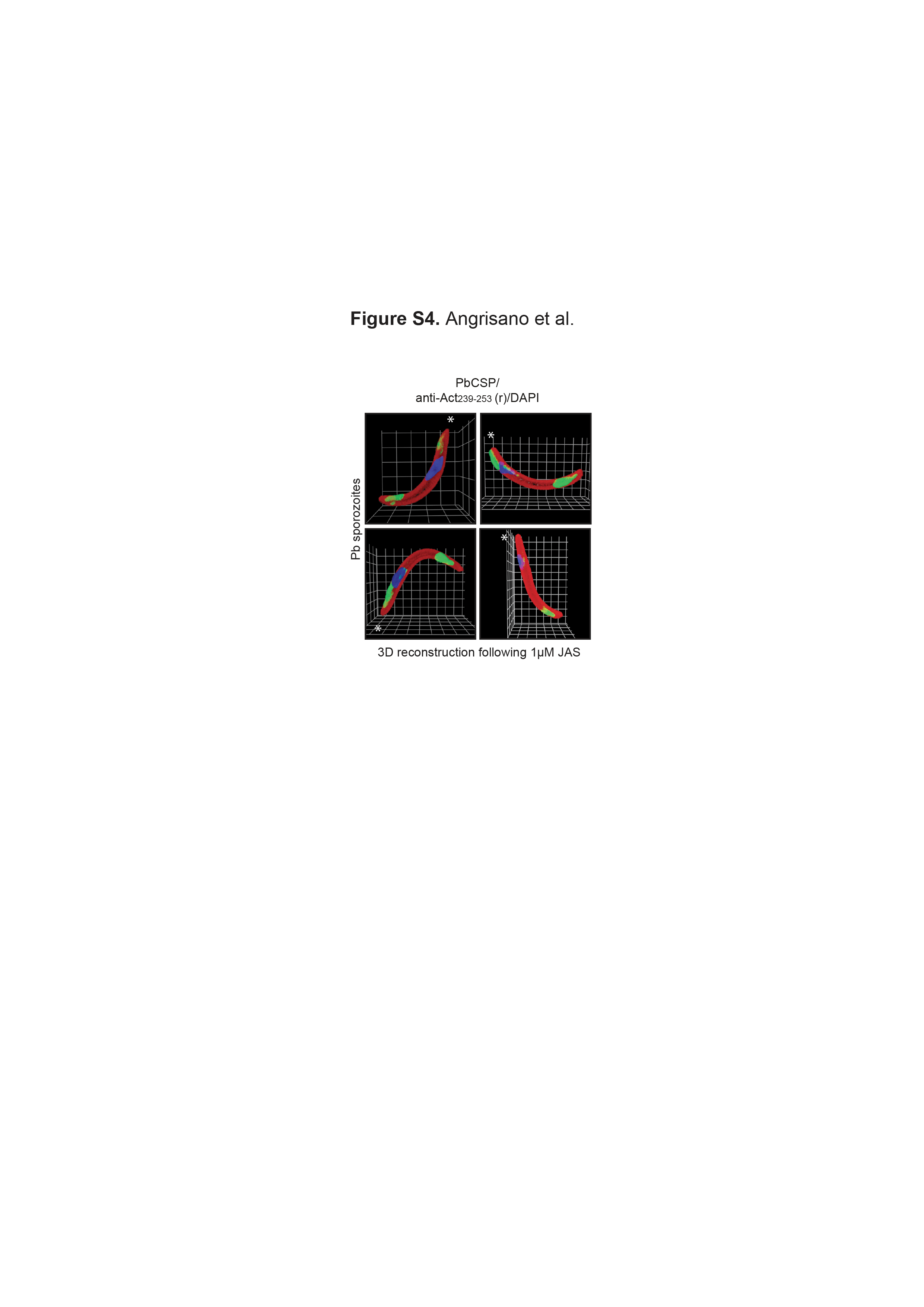

Supplement: Figure S4 — 3D reconstruction of widefield IFA with deconvolution of independent sporozoites treated with 1 µM JAS and labelled with PbCSP (Red), rabbit anti-Act239–252 (Green) and DAPI (Blue). Grid = 1 µm. Asterisk marks parasite apex. See also Movie S1. Gamma settings were altered in 3D reconstructions. (TIF) [file pone.0032188.s004.tif]
